# eHealth Tools That Assess and Track Health and Well-being in Children and Young People: Systematic Review

**DOI:** 10.2196/26015

**Published:** 2022-05-12

**Authors:** Elizabeth Stewart, Alyssa Milton, Hannah Frances Yee, Michael Jae Song, Anna Roberts, Tracey Davenport, Ian Hickie

**Affiliations:** 1 Brain and Mind Centre, The University of Sydney Sydney Australia; 2 Department of Psychiatry, The University of British Columbia Vancouver, BC Canada

**Keywords:** eHealth, children, young people, health, technology, mobile phone

## Abstract

**Background:**

eHealth tools that assess and track health outcomes in children or young people are an emerging type of technology that has the potential to reform health service delivery and facilitate integrated, interdisciplinary care.

**Objective:**

The aim of this review is to summarize eHealth tools that have assessed and tracked health in children or young people to provide greater clarity around the populations and settings in which they have been used, characteristics of digital devices (eg, health domains, respondents, presence of tracking, and connection to care), primary outcomes, and risks and challenges of implementation.

**Methods:**

A search was conducted in PsycINFO, PubMed or MEDLINE, and Embase in April 2020. Studies were included if they evaluated a digital device whose primary purpose was to assess and track health, focused on children or young people (birth to the age of 24 years), reported original research, and were published in peer-reviewed journals in English.

**Results:**

A total of 39 papers were included in this review. The sample sizes ranged from 7 to 149,329 participants (median 163, mean 5155). More studies were conducted in urban (18/39, 46%) regions than in rural (3/39, 8%) regions or a combination of urban and rural areas (8/39, 21%). Devices were implemented in three main settings: outpatient health clinics (12/39, 31%), hospitals (14/39, 36%), community outreach (10/39, 26%), or a combination of these settings (3/39, 8%). Mental and general health were the most common health domains assessed, with a single study assessing multiple health domains. Just under half of the devices tracked children’s health over time (16/39, 41%), and two-thirds (25/39, 64%) connected children or young people to clinical care. It was more common for information to be collected from a single informant (ie, the child or young person, trained health worker, clinician, and parent or caregiver) than from multiple informants. The health of children or young people was assessed as a primary or secondary outcome in 36% (14/39) of studies; however, only 3% (1/39) of studies assessed whether using the digital tool improved the health of users. Most papers reported early phase research (formative or process evaluations), with fewer outcome evaluations and only 3 randomized controlled trials. Identified challenges or risks were related to accessibility, clinical utility and safety, uptake, data quality, user interface or design aspects of the device, language proficiency or literacy, sociocultural barriers, and privacy or confidentiality concerns; ways to address these barriers were not thoroughly explored.

**Conclusions:**

eHealth tools that assess and track health in children or young people have the potential to enhance health service delivery; however, a strong evidence base validating the clinical utility, efficacy, and safety of tools is lacking, and more thorough investigation is needed to address the risks and challenges of using these emerging technologies in clinical care. At present, there is greater potential for the tools to facilitate multi-informant, multidomain assessments and longitudinally track health over time and room for further implementation in rural or remote regions and community settings around the world.

## Introduction

In 2018, the United Nations Children’s Fund released a report on digital technologies in health [1] alongside its Strategic Plan, 2018-2021, which emphasized the importance of using digital (internet- and mobile-based) technology to facilitate health care for children and young people. At the same time, the Early Childhood Developmental Interventions Review Group for the Lancet issued a report making several recommendations for the improvement of assessments and interventions for children or young people [2]. These recommendations included improving the capacity for services to conduct multidomain and multi-informant assessments, connecting children or young people and families with personalized care options, and using digital solutions within health services to allow for broader-scale change [2]. Together, these reports highlight the emerging need to use digital technologies to enhance the delivery of health care for children or young people and their families.

Over the past decade, there has been a rapid growth in the development of digital tools in the health and well-being space [3]. These tools have served various purposes in health care, with the most common uses among children or young people being to deliver interventions (eg, clinician-assisted evidence-based treatments and self-monitoring and self-care), provide education, and facilitate communication for both consumers and clinicians (eg, telehealth or teleconferencing and online peer support groups) [2,4]. Another more recent use of eHealth has been to facilitate the assessment and triage of children or young people through health services [5-12]. These emerging technologies provide users (ie, clinicians and consumers) with secure, web-based platforms for submitting health data (sometimes automatically via biosensors or wearables) without having to be physically present in a hospital or health clinic. The information can be securely shared with health professionals with expertise in children or young people’s areas of need, allowing them to be triaged to appropriate services and connected with ongoing care [5,7-10,13,14]. Thus, these eHealth solutions differ from existing technologies in that their goal is not to deliver interventions or ongoing treatment but rather to facilitate a connection between consumers and pre-existing health services, allow for routine outcome monitoring, and place the person (or family) at the center of care. Furthermore, although some of these tools provide education resources (eg, fact sheets) or communication pathways (eg, web-based chats), they do this with the goal of triaging children or young people to appropriate care.

The literature on eHealth tools that assess and track health outcomes in children or young people is still in its infancy; however, a growing number of studies have reported on such devices over the past decade [4,15]. These tools differ in their health focus (eg, infectious diseases and mental health) [16,17] and locations in which they have been used (ie, rural or urban areas, high- or low-income countries, and specific health settings) [7,18-20]. There has also been variability in terms of the respondent who enters data into the tool (ie, clinician and consumer), the type of data (ie, questionnaires and physiological data), whether the tools have facilitated only assessment or assessment and tracking, and whether they have connected children or young people to clinical care. Given this variability, the specific features of eHealth tools, as well as their efficacy for improving health outcomes and clinical care delivery for children or young people, remain unclear.

Despite the potential benefits of eHealth solutions for children or young people, numerous challenges have been documented in their development, implementation, and uptake among other groups [10,13,21-24]. To be successful, the technologies must be user-friendly, engaging, and accessible to diverse populations. Issues of language, literacy, and culture have all been found to affect accessibility, uptake, and the quality of data [12,21,22,25]. The validity and integrity of data also depend on the availability of appropriately trained health care workers to enter or interpret information, emphasizing the importance of developing and evaluating these tools within the contexts in which they will be used. Finally, issues of privacy, confidentiality, and security are paramount to ensuring that the tools respect the rights of users and are likely to affect the uptake of these technologies [22,25,26].

To our knowledge, no comprehensive reviews have been conducted to examine the efficacy of eHealth tools that assess and track health outcomes in children or young people. As such, it is unclear in which health domains and settings these tools may have the potential to shape clinical care and, importantly, whether their use has been associated with improved health outcomes for children or young people. There is also a need to identify potential challenges and risks of using eHealth tools to ensure that best practice methods are established and consistently used [1,27]. Understanding the available eHealth solutions and their efficacies is critical for shaping future research and development efforts and ensuring efficient expansion of knowledge in this field.

The aim of this systematic review is to summarize eHealth tools that have been developed to assess and track health in children or young people to provide greater clarity about (1) the populations and settings in which these tools have been used (ie, locations, languages, and age groups); (2) characteristics of the tools (ie, health domains assessed, respondent, type of data, presence of tracking, and connection to care); (3) primary outcomes of the study, including whether the use of the tool has been associated with improved health outcomes; and (4) risks and challenges identified during implementation and evaluation.

## Methods

### Overview

The term eHealth has been variously defined in the literature. According to a systematic review, 51 unique definitions have been used for the term, without a clear consensus on a single definition, and the definitions differ in how inclusively they are conceptualized [28,29]. We have chosen to use a definition based on the conceptualization of eHealth offered by Vegesna et al [30] because of its relevance and consistency with the overarching aims of this review; digital technologies are thus defined as noninvasive digital devices that have been used to assess and track the health of a patient or consumer. We used the World Health Organization’s definition of childhood and youth as the period spanning birth to 24 years, whereby children are aged 0 to 9 years, and young people are aged 10 to 24 years [31].

### Search Strategy

The search was conducted according to the PRISMA (Preferred Reporting Items for Systematic Reviews and Meta-Analyses) guidelines [32]. PsycINFO, PubMed or MEDLINE, and Embase were searched via OVID by 3 members of the research team (ES, HY, and AR) on April 27, 2020. The following terms were used ((child*) OR (adolescen*) OR *(young person)* OR (infan*)) AND *((wellbeing)* OR *(health))* AND *(((digital tool)* OR *(digital* AND *tool))* OR *(eHealth)* OR *((mobile application)* OR *(mobile* AND *application)))*. A wildcard (*) was placed at the end of each applicable search term to ensure that all relevant terms were captured. All Medical Subject Heading terms were explored to broaden the search for relevant studies. Date limits were not set on any of the database searches. The reference lists of relevant reviews and identified empirical studies were searched to identify further studies, as per the ancestry method.

### Study Selection Criteria

Studies were included if they met the following criteria:

1. Included children and young people (birth to the age of 24 years) or their parents or carers, as per the World Health Organization’s definition [31]
2. Evaluated a digital device, including internet- or mobile-based technology (ie, noninvasive digital devices, including internet- or mobile-based e-tools and wearable devices), the primary purpose of which was to assess or track the health of the child or young person
3. Focused on a domain of health
4. Evaluation studies, meaning the authors evaluated some aspects of the digital device, including effectiveness, validity, or feasibility; we included all or any type of evaluation studies, which were categorized according to the Center for Disease Control definition (ie, formative, process, and outcome) [33] and National Health and Medical Research Council criteria for study design [34]
5. Reported original research
6. Published in English in a peer-reviewed journal and included human participants

Studies were excluded if they had the following characteristics:

1. Included adults only with no child or young person focus or if >25% of participants were outside our age criteria (birth to the age of 24 years)
2. Evaluated a digital device that was primarily an interventional tool (ie, clinician-led and self-management tools), an educational device (eg, an e-course), a communication device (ie, assistive communication with images or written or spoken language; and teleconferencing only without additional assessment or tracking functionality), or digital technology that did not use internet or mobile technology (eg, electronic medical record systems)
3. Reported results from development or description of the tool that had not yet been evaluated (eg, protocol papers)

### Procedure

**Figure 1:**
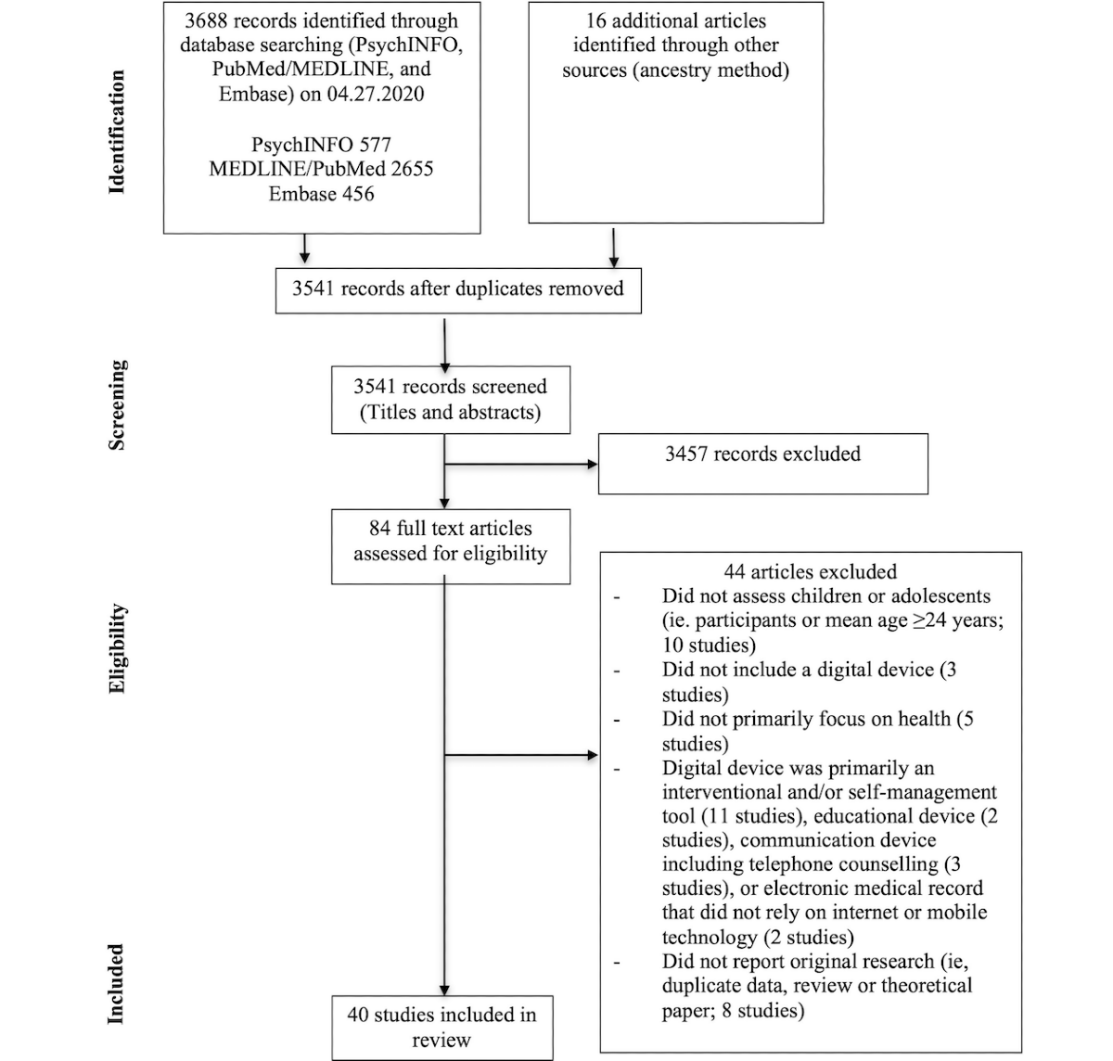
Flow diagram of identification and selection of studies.

Figure 1 displays the process of study selection. The search retrieved 3688 papers, and an additional 16 papers were identified by searching the reference lists of relevant papers and reviews. Of these papers, 95.59% (3541/3704) remained after duplicates were removed. The titles and abstracts of all papers were screened by 2 reviewers (ES and HY). Of the 3541 papers, 84 (2.37%) full-text papers met the inclusion criteria and were obtained. A conservative approach was taken to ensure that relevant papers were not missed, and full-text papers were reviewed if the reviewers could not determine with certainty whether the inclusion criteria were met. The manuscripts of these 84 papers were reviewed by 2 independent raters (ES and HY), and discrepancies were resolved via discussion. Of the 84 papers, 45 (54%) papers were excluded, leaving 39 (46%) papers that were included in the review.

### Data Extraction

The following information was extracted from each paper:

1. Name of the first author, year of publication, and age range of children or young people
2. Language or languages used in the digital application
3. Location where the study was conducted: country, locality (urban or rural), and setting; locality was defined according to the Organization for Economic Co-operation and Development harmonized definition of global urbanization, which uses the population density of the area, that is, rural (<5000 inhabitants) or urban (≥5000 inhabitants) [35]; some studies were conducted in multiple locations, which was considered in categorizing study locality as urban, rural, or a mixture of urban and rural settings
4. Characteristics of the digital tool: health domain assessed, respondent (parent or caregiver, child or young person, clinician, trained health worker, and other), device type (mobile, desktop, and tablet), type of data (questionnaire or survey, images, and physiological), whether the tool allowed for tracking over time (ie, data collected at multiple time points), and whether the device facilitated connection to care (ie, linking patients to health care providers or services)
5. Study characteristics: type of evaluation study, defined according to the Center for Disease Control definition of study evaluation types, that is, formative, process, or evaluation [33]; study type: qualitative, quantitative, or mixed methods; and (3) study design, based on the National Health and Medical Research Council guidelines [34]
6. The primary outcome and main findings from the study, including whether the health of the child or young person was measured as an outcome in the study
7. Funding source, categorized as public sector (ie, government, universities, research institutes, and professional associations), commercial or not-for-profit (NFP) organizations; these categories were guided by an Australian Government resource on university research funding (REF)
8. Any documented risks or challenges associated with the use of the eHealth tool

### Data Analysis

Descriptive analyses were used to summarize variables of interest, including health domain, location, language, type of data, intended user, presence of certain features (ie, tracking over time and connection to care), and type of evaluation. Frequency data and percentages were used to examine and compare studies on key outcome measures. This approach to analysis was taken because of considerable variability in study objectives and designs and as most studies reported simple quantitative, descriptive statistics or qualitative findings.

### Quality Appraisal of Studies

To evaluate the methodological quality of the studies, 2 checklists were used. The Downs and Black checklist [36] was completed for quantitative studies, which measures the quality of both randomized and nonrandomized studies evaluating novel health interventions. The National Institute for Health and Care Excellence Quality Appraisal Checklist was completed for studies reporting qualitative findings [37]. Studies reporting both qualitative and quantitative data were appraised using both checklists. A full description of the checklists and scoring criteria is included in Multimedia Appendix 1 [5-9,16-20,38-66].

## Results

### Demographics of Studies

**Figure 2:**
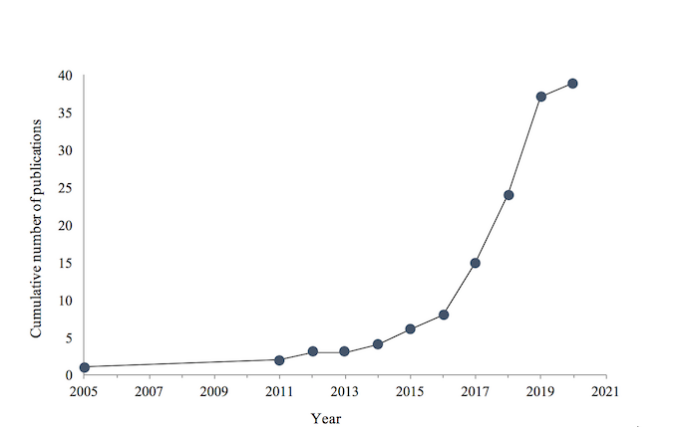
Cumulative number of studies published each year.

Table 1 summarizes the characteristics of the 39 studies included in this review. All (38/39, 97%) but a single (1/39, 3%) study was published in the past decade (2010-2020), and over one-third of the studies (15/39, 39%) were published in the past year (2019-2020; Figure 2). Most studies were conducted in a single country (35/39, 90%), most commonly America (7/39, 18%) or Australia (6/39, 15%). English was the sole language of communication in 49% (19/39) of studies; 13% (5/39) of studies evaluated tools that used English and at least one other language, and 21% (8/39) used languages other than English; the remaining 18% (7/39) of studies did not report enough information to determine which language was used in the tool. Regarding locality, studies were conducted in urban (18/39, 46%), rural (3/39, 8%), or a mixture of urban and rural settings (8/39, 21%); 26% (10/39) studies did not report enough information to determine locality. Digital devices were implemented across 3 main settings: outpatient health clinics (12/39, 31%), hospitals (ie, inpatient units and emergency departments; 14/39, 36%), and community outreach (ie, community spaces that were not formal health clinics; 10/39, 26%) or a combination of these settings (3/39, 8%).

**Table 1:** Demographic characteristics of studies.

Study	Age range of children	Country	Locality^a^	Language used in the device	Setting
Alawna et al, 2019 [50]	19-27 years (mean 22.0)	Turkey	NR^b^	NR	Outpatient health clinic
Binotti et al, 2019 [56]	Infants (age range NR)	Italy	Urban	NR	Hospital
Boyce et al, 2019 [48]	2-59 months	Malawi	NR	English	Outpatient health clinic
Den Boer et al, 2018 [18]	3-17 years	Netherlands	Urban (81%) and rural (19%)	English, German, Spanish, and Dutch	Outpatient health clinic
Detsomboonrat and Pisarnturakit, 2019 [58]	Children in primary school (age range NR)	Thailand	NR	Thai	Community outreach
Dexheimer et al, 2014 [19]	2-18 years	United States	Urban	English	Hospital
Eikelboom et al, 2005 [5]	9 months-16 years	Australia	Rural	English	Outpatient health clinic
Estai et al, 2016 [57]	2-18 years	Australia	Urban	English	Outpatient health clinic
Finocchario-Kessler et al, 2015 [6]	Children (age range NR)	Kenya	Urban (50%) and rural (50%)	English	Hospital
Franke et al, 2018 [20]	18 months-14 years	Ghana	Urban	Twi	Hospital
Galvez et al 2017 [52]	Children (age range NR)	55 countries (worldwide)	NR	English	Hospital
Ginsburg et al, 2015 [16]	Children (age range NR)	Ghana	Urban	English	Outpatient health clinic
Gregory et al, 2017 [39]	<18 years	United Kingdom	Urban	English	Hospital
Han et al, 2019 [53]	13-26 years	China and Australia	Urban	NR	Hospital
Hashemi et al, 2017 [7]	6-18 years	Gaza	Urban	English and Arabic	Community outreach
Heida et al, 2018 [62]	10-19 years	Netherlands	Urban (55%) and rural (45%)	Dutch	Outpatient health clinic
Hussey and Flynn, 2019 [41]	0-21 years	United States	Urban	English	Outpatient health clinic
Iorfino et al, 2017 [8]	16-24 years	Australia	Urban (85%) and rural (15%)	English	Outpatient health clinic
Jeong et al, 2020 [40]	15-19 years	South Korea	Urban	Korean	Hospital, outpatient health clinic, and community outreach
Jiam et al, 2017 [66]	3-22 years	United States	NR	English	Community outreach
Kassam-Adams et al, 2019 [42]	6-14 years	United States	Urban (50%) and rural (50%)	English	Hospital
Kim et al, 2019 [60]	0-5 years	South Korea	NR	NR	Community outreach
Li et al, 2019 [63]	1-18 years	China	Urban	Mandarin	Hospital
March et al, 2018 [17]	5-12 years	Australia	Urban	English	Outpatient health clinic and community outreach
Matin et al, 2020 [59]	0-7 days	Uganda	Rural	Lusoga and English	Community outreach
McCulloh et al, 2018 [49]	0-2 months	United States	NR	English	Hospital
Mohammed et al, 2018 [9]	0-5 years	Ghana	Urban	Twi	Community outreach
Padidar et al, 2019 [64]	0-9 days	Iran	Urban	NR	Hospital
Rath et al, 2018 [45]	0-24 years	Germany	NR	Arab, Farsi, and Russian	Community outreach
Rath et al, 2019 [65]	0-5 years	Germany and Greece	Urban	NR	Hospital
Reid et al, 2011 [43]	14-24 years	Australia	Urban (50%) and rural (50%)	English	Outpatient health clinic
Singh et al, 2017 [51]	0-2 years	India	Urban (85%) and rural (15%)	Hindi, Gujarati, and English	Hospital and outpatient health clinic
Svedberg et al, 2019 [46]	6-13 years	Sweden	Urban (50%) and rural (50%)	Swedish	Hospital
Thabrew et al, 2019 [44]	13-14 years	New Zealand	NR	English	Outpatient health clinic
Thabtah, 2018 [54]	0-17 years	10 countries	NR	11 languages	Community outreach
Thompson et al, 2016 [47]	12-18 years	United States	Urban	English	Outpatient health clinic
Valdes-Angues et al, 2018 [55]	3-18 years	Uganda and United States	Rural	English	Community outreach
van Karnebeek et al, 2012 [61]	0-18 years	Canada	Urban	English	Hospital
Wang et al, 2017 [38]	5-17 years	China	Urban	English and Chinese	Outpatient health clinic

^a^Locality: region in which the eHealth tool was implemented, defined as rural (<5000 inhabitants) or urban (≥5000 inhabitants), according to the Organization for Economic Co-operation and Development’s harmonized definition of global urbanization [35].

^b^NR: not reported.

### Characteristics of eHealth Tools

Table 2 summarizes the characteristics of the studied digital devices.

**Table 2:** Device characteristics.

Study	Health domain	Device	Type of data	Respondent	Tracking over time	Connection to care
Alawna et al, 2019 [50]	General health	Mobile	Physiological	Trained health worker^a^	Unclear	Yes
Binotti et al, 2019 [56]	Developmental	Mobile	Physiological	Trained health worker	No	No
Boyce et al, 2019 [48]	General health	Mobile	Questionnaire or survey	Trained health worker	No	Yes
Den Boer et al, 2018 [18]	Oral health	Mobile	Questionnaire or survey	Clinician^b^ and child or young person^c^	No	Yes
Detsomboonrat and Pisarnturakit 2019 [58]	Oral	Mobile and desktop	Questionnaire or survey	Clinician	No	Yes
Dexheimer et al, 2014 [19]	General health	Desktop	Questionnaire or survey	Clinician	Yes	Yes
Eikelboom et al, 2005 [5]	Ear, nose, and throat	Desktop	Images	Clinician	No	Yes
Estai et al, 2016 [57]	Oral health	Desktop	Images	Clinician and trained health worker	No	No
Finocchario-Kessler et al, 2015 [6]	Infectious	Desktop	Physiological	Parent or caregiver^d^ and trained health worker	Yes	Yes
Franke et al, 2018 [20]	Infectious	Mobile	Questionnaire or survey	Parent or caregiver	No	Yes
Galvez et al, 2017 [52]	Emergency	Mobile	Questionnaire or survey	Clinician	Yes	No
Ginsburg et al, 2015 [16]	Infectious	Mobile	Physiological	Trained health worker	No	Yes
Gregory et al, 2017 [39]	Mental health	Mobile	Questionnaire or survey	Clinician and child or young person	No	Yes
Han et al, 2019 [53]	Vision	Mobile	Physiological	Child or young person	No	No
Hashemi et al, 2017 [7]	Mental health	Desktop and mobile	Questionnaire or survey	Trained health worker	No	No
Heida et al, 2018 [62]	Physical health	Desktop	Questionnaire or survey and physiological	Child or young person and parent or caregiver	Yes	Yes
Hussey and Flynn, 2019 [41]	Mental health	Mobile	Questionnaire or survey	Clinician and child or young person	Yes	Yes
Iorfino et al, 2017 [8]	Mental health	Desktop	Questionnaire or survey	Child or young person	Yes	Yes
Jeong et al, 2020 [40]	Mental health	Mobile	Questionnaire or survey	Clinician and child or young person	No	Yes
Jiam et al, 2017 [66]	Neurological	Desktop	Questionnaire or survey	Parent or caregiver and child or young person	Yes	No
Kassam-Adams et al, 2019 [42]	Mental health	Mobile	Questionnaire or survey	Child or young person	Yes	No
Kim et al, 2019 [60]	Infectious	Mobile	Questionnaire or survey and physiological	Parent or caregiver	Yes	No
Li et al, 2019 [63]	Surgery	Mobile	Questionnaire or survey	Child or young person	No	Yes
March et al, 2018 [17]	Mental health	Desktop, mobile, and tablet	Questionnaire or survey	Clinician, parent or caregiver, education provider, and child or young person	No	No
Matin et al, 2020 [59]	Developmental	Mobile	Questionnaire or survey and physiological	Parent or caregiver	Yes	Yes
McCulloh et al, 2018 [49]	General health	Mobile	Questionnaire or survey	Clinician	No	Yes
Mohammed et al, 2018 [9]	General health	Mobile	Questionnaire or survey	Parent or caregiver	No	Yes
Padidar et al, 2019 [64]	Developmental	Mobile	Physiological images	Clinician and parent or caregiver	No	No
Rath et al, 2018 [45]	General health	Mobile tablet	Questionnaire or survey	Child or young person and parent or caregiver	No	No
Rath et al, 2019 [65]	Infectious	Mobile	Questionnaire or survey	Child or young person	No	No
Reid et al, 2011 [43]	Mental health	Desktop and mobile	Questionnaire or survey	Child or young person	Yes	Yes
Singh et al, 2017 [51]	General health	Desktop and mobile	Questionnaire or survey and physiological	Clinician and parent or caregiver	Yes	Yes
Svedberg et al, 2019 [46]	General health	Mobile	Questionnaire or survey	Child or young person	Yes	Yes
Thabrew et al, 2019 [44]	Mental health	Mobile and tablet	Questionnaire or survey	Child or young person	No	Yes
Thabtah, 2018 [54]	Developmental	Mobile	Questionnaire or survey	Clinician and parent or caregiver	No	No
Thompson et al, 2016 [47]	General health	Desktop	Questionnaire or survey and physiological	Parent or caregiver and young person	Yes	Yes
Valdes-Angues et al, 2018 [55]	Neurological	Desktop and mobile	Questionnaire or survey	Trained health worker	Yes	Yes
van Karnebeek et al, 2012 [61]	Developmental	Desktop, mobile, and tablet	Questionnaire or survey	Clinician	No	No
Wang et al, 2017 [38]	General health and mental health	Mobile	Questionnaire or survey	Clinician, parent or caregiver, and child or young person	Yes	Yes

^a^Trained health workers are staff without professional training who received specific training in the use of the digital tool and associated health domain.

^b^Clinician is defined as a health professional with qualifications in a particular field of practice (including medical doctors and allied health workers).

^c^Child or young person is the individual for whom the eHealth tool was developed.

^d^Parent or caregiver is the primary carer of the child or young person.

### Health Domains

Mental and general health were the most common eHealth domains assessed, with each evaluated in 26% (10/39) of studies. Other health domains assessed included child development (5/39, 13%), infectious diseases (5/39, 13%), oral health (3/39, 8%), neurological illnesses (2/39, 5%), ear nose and throat (1/39, 3%), emergency medicine (1/39, 3%), physical health (1/39, 3%), vision (1/39, 3%), and pediatric surgery (1/39, 3%). A single study assessed multiple health domains (mental and general health) [38]. Given that mental health was more commonly assessed than other health domains, we examined these studies further to determine their aim or purpose and the type of information collected. Of the 23% (9/39) of studies that solely assessed mental health, 33% (3/9) focused on suicide prevention [8,39,40], 22% (2/9) focused on early intervention and prevention of mental illness [7,17], and 44% (4/9) focused on multidimensional assessment or management of mental health symptoms [41-44]. Of the 23% (9/39) of studies that solely assessed general health, 22% (2/9) focused on symptom detection and monitoring [9,45], 22% (2/9) provided a platform for patients to view and monitor their health information [46,47], 33% (3/9) focused on digitalized tracking of clinical decision-making [19,48,49], and 22% (2/9) were primarily for assessment [50,51].

### Data Collection: Respondent, Type of Data, and Device

All devices measured the health of a child or young person; however, devices differed in the person who entered the health information (ie, the respondent: child or young person, parent or caregiver, clinician, and trained health worker). Under half of the devices collected information from multiple respondents (16/39, 41%); other tools collected information solely from a child or young person (8/39, 21%), clinician (6/39, 15%), trained health worker (6/39, 15%), or parent or caregiver (3/39, 8%). Approximately 15% (6/39) of studies collected data in multiple forms (ie, questionnaire or survey, physiological data, or images); otherwise, data were collected solely in the form of questionnaires or surveys (26/39, 67%), physiological data (5/39, 13%), or images (2/39, 5%). Most eHealth tools (31/39, 80%) were configured to collect data on a mobile phone, of which some (9/39, 23%) were also configured to collect data on another device (ie, desktop or tablet).

### Device Features: Health Tracking and Connection to Care

Just under half of the devices tracked children’s health over time (16/39, 41%), and two-thirds (25/39, 64%) connected children or young people to clinical care, whereas the remainder did not.

### Outcome Evaluation: Primary Outcome Measures and Findings

Table 3 summarizes the sample size, type of evaluation, study type and design, and primary outcomes, and a more detailed description of the main findings for each study is presented in Multimedia Appendix 1 (see Table S1). The sample sizes ranged from 7 to 149,329 participants (median 163, mean 5155). Most studies were formative (20/39, 51%) or process (11/39, 28%) evaluations, with fewer outcome evaluation studies (8/39, 21%). Just over one-third of the studies (14/39, 36%) assessed the health of children or young people as either a primary or secondary outcome; however, only a single (1/14, 7%) study assessed whether using the digital tool improved the health of children or young people [43]. This study examined whether the use of *Mobiletype*, an eHealth tool that allowed general practitioners and young people to monitor symptoms of mood, stress, and daily activities in general practice, was associated with improved mental health outcomes compared with treatment as usual. The authors found that use of the device was associated with a significant improvement in emotional self-awareness but found no changes in symptoms of depression, anxiety, or stress; post hoc analyses showed enhanced mental health care at the initial assessment among general practitioners using the tool compared with those who did not.

**Table 3:** Outcomes of studies.

Study	Sample (N)	Type of evaluation^a^	Study type	Study design^b^	Health as outcome^c^	Primary outcome	Challenges or risks of using the tools
Alawna et al, 2019 [50]	58	Formative	Quantitative	Descriptive study	No	Reliability (intra- and interrater reliability)	Clinical utility: questionable accuracy of readings in people with certain health conditions (eg, obesity and limb deformity)
Binotti et al, 2019 [56]	40	Formative	Quantitative	Descriptive study	No	Concordance rating^d^	Clinical safety: partial overestimation of heart rate when <60 beats per minute
Boyce et al, 2019 [48]	799	Process	Mixed methods	Quasi-experimental	No	Efficacy	Accessibility: hardware and software issues (eg, uploading data) Uptake: time consuming
Den Boer et al, 2018 [18]	653	Formative	Mixed methods	Descriptive study	Yes	Usability and efficacy	Accessibility: slow internet connection UX^e^: buttons lacked visual response to input Sociocultural: parents or carers said questions about smoking for children aged 6-11 years were inappropriate and insulting
Detsomboonrat and Pisarnturakit 2019 [58]	441	Formative	Quantitative	Descriptive study	No	Acceptability and efficacy	Accessibility: poor internet connection for some users
Dexheimer et al, 2014 [19]	13,896	Outcome	Quantitative	RCT^f^	No	Efficacy (time from triage to clinical decision)	Clinical utility: clinicians were already implementing best practice guidelines and conducting education without the eHealth tool
Eikelboom et al, 2005 [5]	66	Formative	Quantitative	Descriptive study	No	Concordance rating	Data quality: poor image quality Clinical safety: using eHealth tool alone (without input from a qualified clinician) could result in inaccurate diagnosis and treatment
Estai et al, 2016 [57]	126	Formative	Quantitative	Descriptive study	No	Concordance rating	Data quality: poor image quality
Finocchario-Kessler et al, 2015 [6]	NR^g^	Outcome	Mixed methods	Cross-sectional study	No	Feasibility and efficacy	Accessibility: slow internet connection in some regions Language proficiency or literacy: some users unable to use the tool because of low literacy levels Privacy: concerns about the privacy of data Clinical safety: high turnover of health care workers requiring continuous retraining of staff or risk of inaccurate use of the tool
Franke et al, 2018 [20]	237	Process	Quantitative	Cross-sectional study	No	Concordance rating	Clinical utility: data only entered by parent or caregiver and mostly in binary (yes or no) format; information from clinician said to be important but not possible as multi-informant assessment not available
Galvez et al, 2017 [52]	1252	Process	Quantitative	Descriptive study	No	Use and uptake	Accessibility: only available in countries with internet access and where Google was not blocked
Ginsburg et al, 2015 [16]	7	Formative	Mixed methods	Descriptive study	No	Usability and acceptability	UX: buttons difficult to navigate, pop-ups distracting, difficulty launching application and recording results, too text heavy or more images needed
Gregory et al, 2017 [39]	76	Formative	Quantitative	Descriptive study	No	Feasibility of uptake	Uptake: lower than expected uptake by young people
Han et al, 2019 [53]	150	Outcome	Quantitative	Cohort study	Yes	Validity and reliability	Data quality: mobile phones with low resolution may not clearly show results
Hashemi et al, 2017 [7]	986	Outcome	Quantitative	Descriptive study	Yes	Feasibility	Clinical safety: efficacy of the tool in screening for psychological symptoms not yet validated
Heida et al, 2018 [62]	170	Outcome	Mixed methods	RCT	Yes	Efficacy	Uptake: clinicians not adequately prepared for changes in traditional ways of working and reluctant to enter data twice
Hussey and Flynn, 2019 [41]	56	Formative	Mixed methods	Comparative study with historical control group	No	Use and efficacy	UX: many features needing improvement (eg, emergency alert button, survey tool, SMS text messaging, and notifications)
Iorfino et al, 2017 [8]	232	Process	Quantitative	Nonrandomized experimental trial	Yes	Efficacy	Clinical utility or safety: efficacy for individuals with low to moderate suicidality not studied
Jeong et al, 2020 [40]	13	Formative	Mixed methods	Descriptive study	No	Feasibility, acceptability, and usability	Accessibility: health professionals unable to use the tool because of inadequate training
Jiam et al, 2017 [66]	7	Process	Qualitative	Descriptive study	No	Usability	Language proficiency or literacy: information beyond children’s comprehension capacity and literacy levels
Kassam-Adams et al, 2019 [42]	167	Process	Quantitative	Descriptive study	No	Acceptability and efficacy	Accessibility: lower-income families could not use the tool because of the cost of mobile data
Kim et al, 2019 [60]	149,329	Process	Mixed methods	Descriptive study	No	Uptake, usability, and efficacy	Uptake: of the 3 countries where the tool was implemented, uptake was only seen in Korea and not China or Japan Accessibility: only users with a smartphone could use the eHealth tool Clinical utility: question as to whether increased rates of influenza signaled a local outbreak or new interest in using the tool
Li et al, 2019 [63]	137	Outcome	Quantitative	Pseudo-RCT	Yes	Utility and efficacy	NR
March et al, 2018 [17]	18	Formative	Mixed methods	Descriptive study	No	Feasibility and acceptability	NR
Matin et al, 2020 [59]	18	Formative	Quantitative	Pretest–posttest case series	No	Feasibility and acceptability	Clinical utility: did not assess parents’ accuracy in identifying symptoms aided by the tool; outside of the research study, parents may not receive the same on-call support Clinical safety: only 1 parent attached the wearable band correctly, leading to many incorrect recordings UX: device lacked notifications to encourage care seeking when necessary
McCulloh et al, 2018 [49]	3805	Formative	Mixed method	Descriptive study	No	Use or uptake and usability	NR
Mohammed et al, 2018 [9]	1446	Formative	Quantitative	Descriptive study	Yes	Feasibility and concordance ratings	Accessibility: poor internet connection in some areas; low ownership of mobile phones Data quality: incomplete data entered by some parents
Padidar et al, 2019 [64]	113	Formative	Quantitative	Descriptive study	Yes	Efficacy (concordance rating)	NR
Rath et al, 2018 [45]	405	Formative	Mixed methods	Descriptive study	Yes	Usability and efficacy	Clinical utility: the anonymity of users prevented verification of health conditions and initiation of follow-up care
Rath et al, 2019 [65]	1615	Formative	Quantitative	Cohort study	Yes	Efficacy	NR
Reid et al, 2011 [43]	163	Outcome	Quantitative	RCT	Yes	Change in mental health status	NR
Singh et al, 2017 [51]	16,490	Process	Quantitative	Descriptive study	Yes	Feasibility	Language proficiency or literacy: many parents could not read English messages (Hindi translations integrated to address this issue) Uptake: clinicians and parents were initially resistant to use the new digital system Data quality: errors in data entry related to free text input Clinical utility: customization of question sets needed depending on user characteristics
Svedberg et al, 2019 [46]	46	Process	Qualitative	Descriptive study	No	Feasibility and acceptability	Uptake: low uptake because of required organizational restructuring and competing workplace demands (eg, high workload) UX: software issues related to printing reports and unwanted termination of sessions
Thabrew et al, 2019 [44]	129	Formative	Mixed methods	Pseudo-RCT	No	Efficacy and acceptability	Accessibility: some internet connection issues Language proficiency or literacy: information beyond the comprehension and literacy levels of some low socioeconomic groups
Thabtah, 2018 [54]	1452	Outcome	Quantitative	Descriptive study	Yes	Feasibility and efficacy	NR
Thompson et al, 2016 [47]	937	Process	Quantitative	Descriptive study	No	Use and uptake	NR
Valdes-Angues et al, 2018 [55]	326	Process	Mixed methods	Descriptive study	No	Feasibility	Accessibility: poor internet connection; power cuts; inability to recharge device; slow upload speed of data Data quality: errors in data entry
van Karnebeek et al, 2012 [61]	15	Formative	Qualitative	Descriptive study	Yes	Feasibility and acceptability	Clinical utility: additional features needed to add value to standard care (eg, entering differential diagnosis and accessing databases with medical information)
Wang et al, 2017 [38]	31	Formative	Qualitative	Descriptive study	No	Usability	NR

^a^Type of evaluation defined as follows: (1) formative evaluation: assessed feasibility, appropriateness, or acceptability of the digital device before full implementation; (2) process evaluation: assessed whether the digital device had been implemented as intended; (3) outcome evaluation: measured the effectiveness of the digital device by assessing progress in primary outcomes [33].

^b^Study design based on the National Health and Medical Research Council guidelines: randomized controlled trials (RCTs); pseudo-RCTs; comparative studies with concurrent controls, including nonrandomized experimental trials, cohort studies, case–control studies, or interrupted time series with a control group; comparative studies without a control group, including historical control studies, ≥2 single-arm studies or interrupted time series without a parallel control; case series with either posttest or pre- and posttest outcomes; descriptive studies; or other [34].

^c^Whether an aspect of the child’s or young person’s health was measured as a primary or secondary outcome of the study.

^d^The amount of agreement between the digital tool and clinician ratings.

^e^UX: user experience (user interface or design aspects of the device).

^f^RCT: randomized controlled trial.

^g^NR: not reported.

### Challenges and Risks Identified in Studies

Table 3 summarizes the challenges and risks of using the tools identified in each study. Of the studies examined, most (30/39, 77%) identified at least one challenge or risk, which was related to accessibility (11/39, 28%), clinical utility (9/39, 23%) or clinical safety (5/39, 13%) of the tool, uptake by users (6/39, 15%), data quality (6/39, 15%), user interface or design aspects of the device (user experience; 5/39, 13%); language proficiency or literacy barriers (4/39, 10%), sociocultural barriers (1/39, 3%), and privacy concerns (1/39, 3%). More specifically, accessibility problems were related to poor internet connection, inability to recharge devices because of power cuts, slow or inefficient upload of information, lack of access to a device, and low technological literacy of end users. Clinical utility and clinical safety concerns were related to the validity of data among people with different health conditions, lack of appropriate training of staff, input from a health care professional rather than entirely self-report data to ensure safe and accurate interpretation of results, whether the tool added value over and above standard clinical care, and the safety of tools that had not yet been validated to detect clinical symptoms. Uptake of tools was a frequently cited barrier; however, there was often no further investigation or explanation as to why uptake was lower than expected. Data quality concerns were centered on inaccurate or incomplete data entry (because of human or computer error) and poor-quality images. User experience or design barriers referred to the eHealth tool lacking the necessary features to make it functional and usable for end users. Language proficiency or literacy barriers were centered on users lacking the comprehension and literacy levels to understand and take action from the presented information; this was a concern reported when end users were children, non–English speaking, or from lower socioeconomic backgrounds. Sociocultural barriers were related to the appropriateness of questions and the risk of causing offense or harm.

### Research Funding

Studies were financially supported by the public sector (ie, government, universities, research institutes, and professional associations) and commercial or NFP organizations (28/39, 72%). Receiving funding from ≥1 sector was the most common (16/39, 41%), followed by funding solely from public sources (6/39, 15%) and NFPs (5/39, 13%). No study was funded solely by the commercial sector; however, commercial funding contributed to nearly one-third of studies with combined funding sources (5/16, 31%). The remainder of the studies did not receive external financial support or did not report it in the paper (11/39, 28%).

### Quality Ratings of Selected Papers

The methodological quality of the Downs and Black checklist was rated for 95% (35/39) of studies that included quantitative data: 64% (25/39) of studies had a low chance of bias, 36% (14/39) of studies had a moderate chance of bias, and no studies had a high chance of bias (see Table S2, Multimedia Appendix 1). The National Institute for Health and Care Excellence Quality Appraisal Checklist was completed for 44% (17/39) of studies that included qualitative data: 59% (10/17) of studies received a maximum score of 2 for quality, and 41% (7/17) of studies received a partial score of 1; no studies received a score of 0 (see Table S3, Multimedia Appendix 1).

## Discussion

### Principal Findings

The aim of this systematic review was to summarize eHealth tools designed to assess and track health outcomes in children and young people to clarify the current scope, nature, and efficacy of this emerging type of technology in health care. Our findings revealed exponential growth in the development and evaluation of these tools over the past 10 years; however, the results showed that the research is still in its infancy, with most studies assessing feasibility, acceptability, usability, or uptake of a device rather than the efficacy of tools in relation to health outcomes. Overall, the current tools showed potential to enhance the assessment and tracking of children or young people in health services around the world. Further research is needed to evaluate the efficacy of tools for improving health outcomes and clinical care delivery, as well as to identify and address the risks and challenges of implementing these tools as part of standard clinical care.

There are numerous potential advantages of using eHealth solutions for children and young people, including the ability to conduct multidomain and multi-informant assessments, undertake continuous monitoring, and assist with timely connection to personalized clinical care [1,2,13]. Encouragingly, over half of the tools facilitated a connection between the child or young person and a health care provider; however, less than half tracked children’s health data over time or collected information from multiple informants (ie, child or young person, parent or caregiver, and health care professional). These findings demonstrate an untapped potential of eHealth solutions in facilitating multi-informant assessments and longitudinally tracking health over time among children or young people, which is key to achieving comprehensive, multidisciplinary care [2]. In addition, data were most commonly collected in the form of surveys or questionnaires, illustrating a lag in uptake and integration of newer technologies (eg, biosensors or wearables to collect physiological data). Such technology has the potential to enhance symptom detection and clinical decision-making [13,14,19,48,49] and may be an important area for future research to explore.

Another potential advantage of eHealth is its ability to overcome geographical, financial, and social barriers that hinder the provision of health services in specific populations and locations [10,67]. A small number of studies evaluated devices that were implemented across multiple countries [45,52-55], highlighting the ability of digital technologies to provide health care with greater reach. However, fewer tools were implemented in rural areas compared with urban areas, and there was less implementation in community outreach settings compared with hospitals and health clinics. Although using eHealth tools in health clinics and hospitals is a step forward from traditional paper-based methods in terms of data management and integrated care, there is greater potential for the tools to engage hard-to-reach populations in regional and community settings [9,55]. The higher percentage of devices used in health clinics and hospitals may be as community settings do not always incorporate systematic health tracking into their procedures or reflect a lack of availability of skilled health professionals to collect and enter health information in community settings. A number of studies overcame this issue by using trained health workers (ie, staff who received specific training in the health condition and digital device but were not specialists in the field); these trained health workers were able to collect information in the community, with studies finding that this did not compromise the validity or reliability of data or clinical care [6,7,48,50,56,57]. Another solution was to collect information solely from the consumer (ie, child or young person or their parent or guardian), which is particularly common in studies examining mental health [8,43,44]. Together, these results demonstrate room for broader implementation in rural or remote regions and community settings around the world. Nevertheless, an important issue to recognize is that rural and vulnerable populations are currently experiencing the largest digital divide [68-70]. Inequalities in access exist because of variations in location, age, education, and income level. For instance, the cost of internet access is higher in rural or remote regions than in urban areas, which is compounded by the fact that some rural residents have less disposable income than their metropolitan counterparts. Thus, to truly overcome geographical, financial, and social barriers and reach these populations, researchers must consider the broader socioeconomic context from which these access issues stem.

The eHealth tools studied focused on various domains of health, including infectious diseases, child development, and neurological conditions; however, the most widely assessed domains were general and mental health. The focus on mental health tools may reflect a growing need and demand for mental health care among children and young people, increasing evidence supporting eHealth in the field of mental health, and increased funding for mental health tools [71,72]. Only one of the eHealth tools assessed multiple health domains [38], despite this often being important for gaining a holistic picture of a child or young person’s health concerns. The development of eHealth tools that assess health multidimensionally is likely to be important in future eHealth tools, perhaps assessing not only current symptoms but also broader social or environmental factors related to the etiology and trajectory of illness and barriers to or facilitators of accessing care [10,13].

Various challenges and risks were identified in relation to the implementation and use of eHealth tools. These barriers were related to the accessibility and functionality of devices, including poor internet connection [6,9,15,18,44,48,52,55,58] and user interface or design aspects of the tool [16,18,41,46,59]. Clinical utility was another barrier identified in papers, mainly relating to the tool lacking features that provided added value to standard care [8,19,45,51,59-61]. User uptake was a challenge, with multiple studies reporting lower than expected uptake and reluctance to use the digital tool; this was an issue reported among diverse user groups, including clinicians, parents or carers, and children or young people [39,46,48,51,59,60,62]. These findings are in line with the Eysenbach [73] law of attrition, which is based on the observation that high participant dropout rates are common in eHealth research focusing on novel digital health tools; although researchers may dismiss or underreport this information, the observation meaningfully reflects the real-world uptake of digital tools currently. Some of the reasons for low uptake included implementation barriers (eg, competing time, modified professional roles, and organizational restructuring) [46,48,62], privacy concerns [6], socioeconomic factors (eg, cost of data) [42], and language proficiency or literacy issues [6,44,51]. Incorporating participatory design (co-design) and user testing methodologies into future protocols may help to understand and address these barriers [10,22]. Data quality was another barrier that was reported, which was related to human error in data entry [51,55] or incomplete data input [9]. A study overcame the issue of human error by minimizing free-text input and using predefined options [51]. Although this is not feasible for all tools, such as when obtaining qualitative health information, it provides a solution for quantitative health data. Sociocultural issues were mentioned in just 1 study; Den Boer [18] reported that parents or carers in some communities found questions about smoking in children aged 6 to 11 years *insulting* and inappropriate. The study researchers justified the inclusion of the questions by saying that they were important and relevant for certain communities or user groups. This raises the issue of whether universal questionnaires can be used in eHealth tools or whether customized question sets need to be developed for the target group. The findings of Singh [51] supported a configurable or individualized approach, with the researchers concluding that individualization was critical to the clinical utility and safety of eHealth tools. Despite studies identifying challenges and risks, there was minimal discussion on how to address the identified issues; moreover, just under a quarter of studies did not report any potential risks or challenges of implementing the device [17,38,43,47,49,54,63-65].

Our review of funding sources, which showed that financial support came from a mixture of public, commercial, and NFP bodies, is unsurprising, as the development and implementation of digital tools often involve the collaboration of professionals from multiple disciplines who belong to different bodies [25,74]. Interestingly, funding from commercial bodies was uncommon. This may reflect the fact that the tools reviewed were in the preliminary stages of research (development and implementation) rather than at a more advanced stage of commercialization, the latter of which we would expect to attract more investment from commercial organizations [74].

### Recommendations for Future Research

The findings of this review demonstrate a clear need for further research into the efficacy and validity of eHealth tools that assess and track health outcomes in children and young people. Future evaluation studies should focus on changes in the health outcomes of users, as well as the clinical care pathways. Further investigation of the risks and challenges of implementing devices is also important, particularly relating to sociocultural factors, language proficiency or literacy, and privacy concerns, as these were seldom mentioned but are likely to affect the clinical utility, safety, and uptake of tools [10,23]. Overall, these findings are consistent with results from a prior systematic review of eHealth solutions in adults, which found a gap between the postulated and empirically demonstrated benefits of eHealth technologies, a lack of robust research trials into validity and efficacy, and inadequate investigation of risks or challenges of using these technologies in health care [75]. This review has uncovered several features of eHealth tools that may facilitate comprehensive assessments and integrated care in future technologies:

1. Capacity for multi-informant assessment, including input from a health professional and the child or young person or their parent or caregiver
2. Multidomain assessments, allowing for a holistic picture of the child or young person’s health to be captured rather than assessing health in one domain
3. Tracking over time (ie, capacity and use of tools for assessment at multiple time points)
4. Configurability of question sets or content depending on characteristics (eg, demographic, sociocultural, and health concerns) of the target group
5. Connection to clinical care that is tailored to the child or young person’s current needs
6. Trialing integration of newer technologies (eg, biosensors or wearables to collect physiological data) for relevant health domains

### Limitations

Although this review provides important insights into a novel field of eHealth, the conclusions that can be drawn about the efficacy and validity of eHealth solutions are limited as most studies were formative and process evaluations that assessed feasibility, acceptability, usability, or uptake of a device. Outcome evaluation studies were rare, with just 3 randomized controlled trials conducted to date. These early phase research studies are necessary precursors to more rigorous validity and efficacy studies; however, they need to be followed by more thorough evaluation studies to determine whether the tools are effective in improving health outcomes and clinical care. We limited our search to studies published in English, which may have biased our results. Furthermore, although the strength of this review is that it presents the state of eHealth tools for supporting health in children or young people, it inevitably fails to consider the immense variation that lies within each health domain. Our search strategy was not without limitations. We did not include all relevant terms (eg, internet and technology) as the inclusion of these broader terms returned >15,000 articles, which was not considered realistic for screening. Nevertheless, we believe that the search strategy balanced scientific rigor and feasibility and was sufficiently rigorous to pick up relevant articles. Finally, this paper was not preregistered with PROSPERO; however, the search strategy remained the same over time.

### Conclusions

eHealth tools that assess and track health outcomes in children or young people and connect individuals with personalized care options have enormous potential in health services around the world. Many of the existing tools are in the early stages of pilot and feasibility testing; however, the literature is promising in the potential to use these tools in future clinical care. Further research is needed to evaluate the validity and efficacy of these eHealth tools and investigate the potential risks and challenges of implementation as part of standard clinical care. With future research and development efforts in place, these tools have the potential to facilitate collaborative decision-making, improved communication, transmission of remote health data, and real-time assessment and tracking and take a positive step forward in digitalizing health practices.

## Appendix

Multimedia Appendix 1 Digital review.

## Abbreviations

NFP: not-for-profit
PRISMA: Preferred Reporting Items for Systematic Reviews and Meta-Analyses
