# Supplementary material for: eHealth Tools That Assess and Track Health and Well-being in Children and Young People: Systematic Review
Source: J Med Internet Res. 2022 May 12;24(5):e26015. doi: 10.2196/26015 (PMC9136648; doi:10.2196/26015)
Supplement: Multimedia Appendix 1 [file jmir_v24i5e26015_app1.docx]

Multimedia Appendix 1

Table S1. Brief description of primary outcomes findings

| First author | Year | N | Primary outcome | Findings |
| --- | --- | --- | --- | --- |
| Alawna | 2019 | 58 | Reliability (inter- and intra-rater reliability) | The smartphone goniometer record (SGR) application and the traditional universal goniometer demonstrated excellent inter- and intra-rater reliability to measure ankle dorsiflexion and plantarflexion. |
| Binotti | 2019 | 40 | Concordance rating (agreement between set and communicated heart rate) | The NeoTapAdvancedSupport (NeoTapAS) mobile application showed good agreement with communicated heart rate (Cohen's kappa=0.80), although showed partial overestimation of heart rate when below 60 beats per minute. |
| Boyce | 2019 | 799 | Efficacy (assessing and classifying illness in children using the digital tool vs. paper-based methods) | Using the integrated Community Case Management (iCMM) mobile application enhanced assessment and classification of childhood illnesses among health care workers (81% correct classification using the tool vs. 58% correct using paper-based methods). When broken down into different illnesses/symptoms, improved accuracy when using the tool was only seen in some domains (e.g. cough, malnourishment, oedema), and not others (e.g. fever, diarrhoea, convulsions) |
| Den Boer | 2018 | 653 | Usability and efficacy | The eHealth tool was rated as user-friendly by both patients and dentists and allowed for timely collection of oral health data (usability). The data collected was in keeping with population based prevalence data for dental caries (efficacy). |
| Detsomboonrat, | 2019 | 441 | Acceptability and efficacy | Oral Health Survey Mobile Application (OHSMA) was rated by participants as less acceptable than traditional paper forms for inputting and recording oral health data, but more effective for searching and reporting these data. The tool was effective in accurately identifying dental carries in schoolchildren. |
| Dexheimer | 2014 | 13, 896 | Efficacy (time from triage to clinical decision) | No significant difference (p>0.05) between using the eHealth tool and standard care in time to clinical decision, hospital admission rate or emergency department length of stay. |
| Eikelboom | 2005 | 66 | Concordance rating | Significant agreement (p<0.001) on assessment, diagnosis and referral recommendations between medical specialists and the eHealth tool. |
| Estai | 2016 | 126 | Concordance rating | Accurate and reliable diagnosis using the eHealth tool, with high inter-examiner agreement between the eHealth tool, oral health professionals (non-dentists) and dentists (Kappa= 0.82–0.88). |
| Finocchario-Kessler | 2015 | NR | Feasibility and efficacy | The HIV Infant Tracking System (HITSystem) was successfully implemented into 10 low-resource health settings, and showed good uptake by clinicians. There was a higher rate of retention/retesting of children with HIV in clinics using tool compared to national averages (without the tool) |
| Franke | 2018 | 237 | Concordance rating | Good agreement between the eHealth tool (rated by parents) and clinician ratings on detection of symptoms of cough, fever, diarrhoea, and vomiting (kappa=0.42–0.59) but weak agreement for treatment recommendations (kappa=0.11). |
| Galvez | 2017 | 1252 | Utilisation and uptake (number of user sessions and countries where it was downloaded) | Over a 3 month period, Pedi Crisis was used by 1252 users in 108 countries, demonstrating promising global uptake and utilisation of the device. |
| Ginsburg | 2015 | 7 | Usability and acceptability | Usability of mPneumonia varied depending on participants prior experience with devices and touch-screens, but was positive overall. Clinical content was acceptable. Feedback given on ways to improve user interface. |
| Gregory | 2017 | 76 | Feasibility of uptake | The eHealth tool (BeSafe) was downloaded by 18% of young people in hospital, with 68% of young people saying they would download the tool after discharge, however many did not. Further feasibility testing needed to establish barriers to uptake. |
| Han | 2019 | 150 | Validity and reliability | The Vision at Home tool was accurate and reliable for measuring visual acuity. There was high agreement between the tool and clinician ratings. |
| Hashemi | 2017 | 986 | Feasibility | The eHealth tool was able to be successfully used to screen for psychological symptoms (e.g. post-traumatic stress, depression, somatic symptoms) in children in war torn zones, and identified follow-up treatment where necessary. |
| Heida | 2018 | 170 | Efficacy | Home Telemonitoring using the eHealth tool was as safe and effective as conventional face-to-face follow-up for adolescents with inflammatory bowel disease. Use of the tool was associated with reduced outpatient visits and societal costs compared to conventional follow-up. |
| Hussey | 2019 | 56 | Utilisation and efficacy | Using the Addiction Comprehensive Health Enhancement Support System (ACHESS) was associated with higher completion of treatment among youth with substance use problems (54%) compared to controls (54% vs. 43%) and reduced the number of days in treatment (108.1 vs. 76.8 days); however these differences weren't statistically significant. During focus groups, staff said aspects of the tool positively impacted patient outcomes, while other elements offered little utility. |
| Iorfino | 2017 | 232 | Efficacy | The Synergy Online System was used to successfully detect suicidality among young people and provided clinicians with real-time online alerts. Young people with high suicidality were managed with the suicide escalation protocol, which enabled an appropriate and timely clinical response. |
| Jeong | 2020 | 13 | Feasibility, acceptability and usability | Brake of My Mind (BoMM) mobile application was considered feasible and acceptable for end users (survivors of suicide, and health professionals). There were few usability problems reported by participants. |
| Jiam | 2017 | 7 | Usability | The eHealth tool (Important Information About Me, IIAM) was found to be usable, easy to navigate and useful for communicating and managing health information between parents and health care professionals. However, parents said the tool was not appropriate for use by children with the biggest usability limitation being children’s age and literacy levels. |
| Kassam-Adams | 2019 | 167 | Acceptability and efficacy | Children/young people rated the eHealth tool as easy to use, useful for their recovery, and many said they would recommend it to others. Measures collected by the tool showed strong convergent validity with validated screeners. |
| Kim | 2019 | 149,329 | Uptake, usability and efficacy | The Fever Coach mobile app successfully collected data from and provided care information to a significant portion of the target population. The prevalence data collected by the eHealth tool was significantly correlated with population based data on influenza and modelling data could be used to predict outbreaks in the population. |
| Li | 2019 | 137 | Utility and efficacy | Using the 317-Nursing Mobile Application Program (317NAP) was useful in monitoring short-term recovery of day surgery patients with several advantages to traditional care (e.g. convenience, accessibility, efficiency, low cost). The eHealth tool was associated with faster follow-up time (p<0.05) and better recovery (ns) compared to the control group. |
| March | 2018 | 18 | Feasibility and acceptability | The eHealth tool (Interactive Child Distress Screener, ICDS) demonstrated feasibility and acceptability among participants in terms of the user interface/design and accurate understanding of the emotion and behaviour constructs represented in the tool. Key areas of improvement were identified and changes were made during the study in an iterative process. |
| Matin | 2020 | 18 | Feasibility and acceptability | NeMo mobile app (and wearable sensor) showed acceptability and feasibility of use by mothers in their own home. Mothers frequently used the tool to assess their child, were able to understand how to use it with minimal training, and retained this skill for 6 days. The tool also initiated care-seeking behaviours. All mothers agreed the tool was easy to use and improved knowledge of timely help-seeking behaviours for potentially sick babies. |
| McCulloh | 2018 | 3805 | Utilisation, uptake and usability | Over an 8-month period, the eHealth tool (Electronic Decision Support Tool, ECDS) was downloaded by 3805 individuals in the USA. Health care workers rated the tool as easy to use (94%), and thought future users would be able to learn to use the tool quickly (91%). No user interface or workflow criticisms were identified. |
| Mohammed | 2018 | 1446 | Feasibility and concordance ratings | The eHealth tool (Electronic Health Information and Surveillance System eHISS) was successfully completed by parents and found to be feasible for assessing childhood illness (e.g. fever, diarrheoa). There was moderate to very good agreement between the tool (rated by parents) and clinician ratings of fever, diarrhoea and cough (kappa=0.49–0.89). |
| Padidar | 2019 | 113 | Efficacy (concordance rating; sensitivity and specificity, correlation) | The eHealth tool estimated bilirubin levels in newborns with sensitivity of 68% and specificity of 92%. The tool had a moderate correlation (r=0.479) with total serum bilirubin values. |
| Rath | 2018 | 405 | Usability and efficacy | The Vienna Vaccine Safety Initiative (ViVI) Health Survey enabled rapid and comprehensive anonymous assessment of general health risks, needs and exposures to illness in young migrants, including communicable and non-communicable illness. |
| Rath | 2019 | 1615 | Efficacy | The Vienna Vaccine Safety Initiative Disease Severity Score (ViVI Score) accurately measured influenza and influenza‐like disease severity in patients. The risk adjusted ViVI score was highly predictive of physician prescribing practice for antibiotics and antivirals; however, patients with the same severity score at different sites often did not receive the same treatment. |
| Reid | 2011 | 163 | Change in mental health status | Use of Mobiletype (which allowed monitoring of symptoms of mood, stress and daily activities) was associated with a significant improvement in emotional self-awareness compared to the control condition where only daily activities were monitored; there was no change in symptoms of depression, anxiety or stress using the eHealth tool. Use of the tool also led to enhanced GP mental health care at first assessment. |
| Singh | 2017 | 16,490 | Feasibility | The eHealth tool (Integrated Child Health Record cloud, iCHRCloud) provided a secure and sustainable platform to digitalise real-time child growth and vaccination information and facilitate doctor-parent communication. |
| Svedberg | 2019 | 46 | Feasibility and acceptability | The eHealth tool (Sisom) was successfully implemented in 4 paediatric care centres in Sweden. Facilitators to implementation success included the tool's purpose aligning with organisation's values and goals, staff beliefs on usefulness and usability of the tool, and staff willingness to adapt their professional roles in the tool's implementation within the organisation. Children demonstrated adequate acceptability of the tool's design and purpose. |
| Thabrew | 2019 | 129 | Efficacy (time taken to complete screener, accuracy of detection rate) and acceptability | The YouthCHAT screening tool was twice as fast as the traditional assessment and showed greater detection rates of many psychosocial issues. High school students and health care providers reported overall acceptability of the tool. |
| Thabtah | 2018 | 1452 | Feasibility and efficacy | The eHealth tool (ASDTest) was found to be an easy and time-efficient way to screen for Autism Spectrum Disorder. Scores derived using machine-learning algorithms showed promising results with respect to sensitivity, specificity and accuracy. |
| Thompson | 2016 | 937 | Utilisation and uptake | Uptake and usage of the eHealth tool was similar among young people (12-17 years) and parents of younger children (0-11 years), but differences were observed in the patterns of usage: young people were less likely than parents of young children to review appointments, test results and problem lists, and send messages to their health care providers. |
| Valdes-Angues | 2018 | 326 | Feasibility | The eHealth tool (Magpi) was a feasible and reliable way of collecting medical information from participants and allowed investigators to geographically map health status in children living in rural Uganda over a 12-week period. They identified specific regions where lack of access to medication was common. |
| van Karnebeek | 2012 | 15 | Feasibility and acceptability | The eHealth tool (WebAPP) was a feasible way of enhancing diagnosis and care of children with intellectual disability. Clinicians identified several necessary changes to the tool that would improve acceptability and feasibility of implementation (e.g. search functions by signs/symptoms). |
| Wang | 2017 | 31 | Usability | Parents, children (8-13 years) and clinicians all rated the eHealth tool (PROMIS) as easy to use and age-appropriate, with no concerns about the design interface or usability of the tool raised. The administration portal recorded data accurately. |

**Description of Quality Rating Checklists**

***Downs & Black (1998) Checklist***

The Downs & Black (1998) Checklist contains 27 items, 10 of which were excluded as they are specific to interventional studies, which were not included in this review. The scale yields scores on four subscales assessing quality of reporting (items 1–8), external validity (item 9) and internal validity: statistical and methodological bias (items 10 – 12), and selection bias (items 13 – 17). Each item was scored 0 (no or cannot tell) or 1 (yes). Items 4, 13 and 15 were only relevant to studies comparing two or more groups, e.g. cohort studies, case-control studies or RCTs. Items 7 and 16 (loss of patients at follow-up) were only applicable to longitudinal studies. Articles were rated as having low chance of bias (>/=75% items endorsed), moderate chance of bias (50-75% items endorsed) or high chance or bias (<50% items endorsed). The checklist was chosen for its good test-retest reliability (r=0.88), inter-rater reliability (r=0.75), and internal consistency (Kruder-Richardson formula 20=0.89) (Downs & Black, 1998). The checklist was completed for quantitative and mixed-method studies in our review.

***NICE Quality Appraisal Checklist***

The NICE Quality Appraisal Checklist assesses reporting in qualitative research studies (NICE, 2012). The checklist contains 15 items pertaining to theoretical approach (items 1–2), study design (item 3), data collection (item 4), trustworthiness (items 5–7), analysis (items 8–13), ethics (item 14) and an overall assessment item that is scored based on responses to previous items (item 15). Items are rated on a 3-point scale (e.g. appropriate/ inappropriate/ unsure; clear/ unclear/ mixed). Items 1 to 14 were scored as 0 (inappropriate/unsure) or 1 (appropriate/clear). Item 15 was rated as 0 (few or no checklist criteria fulfilled [<5/14 items], conclusions are likely or very likely to alter), 1 (some criteria fulfilled [5-11/14 items], and where they have not been fulfilled or not adequately described, the conclusions are unlikely to alter) or 2 (all or most criteria fulfilled [≥12/14 items], and where they have not been fulfilled the conclusions are very unlikely to alter). The NICE checklist was included to rate methodological quality of studies that included qualitative findings. The checklist was completed for qualitative and mixed-method studies in our review.

Table S2. Quality assessment of studies included in the review using the Downs & Black Checklist

| Studies | | | | Quality Assessment Criteria from the Downs and Black Checklist | | | | | | | | | | | | | | |  | |
| --- | --- | --- | --- | --- | --- | --- | --- | --- | --- | --- | --- | --- | --- | --- | --- | --- | --- | --- | --- | --- |
| First author. Year | Quality of Reporting | | | | | | | | | External Validity | Internal Validity | | | Selection Bias | | | | |  |  |
|  | 1 | 2 | 3 | | 4 | 5 | 6 | 7 | 8 | 9 | 10 | 11 | 12 | 13 | 14 | 15 | 16 | 17 | Sum | % |
| Alawna. 2019 | 1 | 1 | 1 | | NA | 1 | 0 | N/A | 0 | 0 | 1 | 1 | 1 | N/A | 1 | N/A | N/A | 1 | 9/12 | 75% |
| Binotti. 2019 | 1 | 1 | 1 | | N/A | 1 | 0 | N/A | 0 | 0 | 1 | 1 | 1 | N/A | 1 | N/A | N/A | 1 | 9/12 | 75% |
| Boyce. 2019 | 1 | 1 | 1 | | 1 | 1 | 0 | N/A | 1 | 1 | 1 | 1 | 1 | 0 | 0 | 0 | N/A | 1 | 11/15 | 73% |
| Den Boer. 2018 | 1 | 1 | 1 | | N/A | 1 | 0 | 0 | 0 | 0 | 1 | 1 | 1 | N/A | 1 | N/A | 0 | 1 | 9/14 | 64% |
| Detsomboonrat. 2019 | 1 | 1 | 0 | | N/A | 1 | 1 | N/A | 1 | 0 | 1 | 1 | 1 | N/A | 1 | N/A | N/A | 0 | 9/12 | 75% |
| Dexheimer. 2014 | 1 | 1 | 1 | | 0 | 1 | 1 | N/A | 1 | 1 | 1 | 1 | 1 | 1 | 1 | 0 | N/A | 1 | 13/15 | 87% |
| Eikelboom. 2005 | 1 | 1 | 1 | | N/A | 1 | 0 | N/A | 0 | 0 | 1 | 1 | 1 | N/A | 1 | N/A | N/A | 1 | 9/12 | 75% |
| Estai. 2016 | 1 | 1 | 1 | | N/A | 1 | 0 | N/A | 1 | 0 | 1 | 1 | 1 | N/A | 1 | N/A | N/A | 1 | 10/12 | 83% |
| Finocchario-Kessler. 2015 | 1 | 1 | 0 | | N/A | 1 | 1 | N/A | 0 | 1 | 1 | 1 | 1 | N/A | 0 | N/A | N/A | 1 | 9/12 | 75% |
| Franke. 2018 | 1 | 1 | 1 | | N/A | 1 | 0 | N/A | 1 | 1 | 1 | 1 | 1 | N/A | 1 | N/A | N/A | 1 | 11/12 | 92% |
| Galvez. 2017 | 1 | 1 | 0 | | N/A | 1 | 1 | N/A | 0 | 1 | 1 | 1 | 1 | N/A | 1 | N/A | N/A | 1 | 10/12 | 83% |
| Ginsberg. 2015 | 1 | 0 | 1 | | N/A | 1 | 0 | N/A | 0 | 1 | 0 | 1 | 1 | N/A | 0 | N/A | N/A | 1 | 7/12 | 58% |
| Gregory. 2017 | 1 | 1 | 1 | | N/A | 1 | 0 | N/A | 0 | 1 | 1 | 0 | 1 | N/A | 0 | N/A | N/A | 1 | 8/12 | 67% |
| Han. 2019 | 1 | 1 | 1 | | 0 | 1 | 0 | N/A | 0 | 0 | 1 | 1 | 1 | 0 | 1 | 0 | N/A | 1 | 9/15 | 60% |
| Hashemi. 2017 | 0 | 1 | 1 | | N/A | 1 | 1 | N/A | 0 | 1 | 1 | 1 | 1 | N/A | 1 | N/A | N/A | 0 | 9/12 | 75% |
| Heida,. 2018 | 1 | 1 | 1 | | 1 | 1 | 0 | 0 | 1 | 1 | 1 | 1 | 1 | 1 | 1 | 1 | 0 | 1 | 14/17 | 82% |
| Hussey. 2019 | 1 | 1 | 1 | | 1 | 1 | 0 | 1 | 0 | 0 | 1 | 1 | 1 | 1 | 0 | 1 | 1 | 0 | 12/17 | 71% |
| Iorfino. 2017 | 1 | 1 | 1 | | 1 | 1 | 1 | N/A | 1 | 1 | 1 | 1 | 1 | 0 | 0 | 1 | N/A | 1 | 13/15 | 87% |
| Jeong. 2020 | 1 | 1 | 1 | | N/A | 1 | 1 | N/A | 0 | 0 | 1 | 1 | 1 | N/A | 1 | N/A | N/A | 1 | 10/12 | 83% |
| Jiam. 2017 |  |  |  | |  |  |  |  |  |  |  |  |  |  |  |  |  |  |  |  |
| Kassam-Adams. 2019 | 1 | 1 | 1 | | N/A | 1 | 1 | N/A | 1 | 1 | 1 | 1 | 1 | N/A | 1 | N/A | N/A | 1 | 12/12 | 100% |
| Kim. 2019 | 0 | 1 | 1 | | N/A | 1 | 0 | N/A | 1 | 1 | 1 | 1 | 1 | N/A | 0 | N/A | N/A | 0 | 8/12 | 67% |
| Li. 2019 | 1 | 1 | 1 | | 0 | 1 | 1 | 1 | 1 | 1 | 1 | 1 | 1 | 1 | 1 | 0 | 1 | 0 | 14/17 | 82% |
| March. 2018 | 1 | 1 | 1 | | N/A | 1 | 1 | N/A | 0 | 0 | 1 | 1 | 1 | N/A | 0 | N/A | N/A | 1 | 9/12 | 75% |
| Matin. 2020 | 1 | 1 | 1 | | N/A | 1 | 1 | 1 | 1 | 1 | 1 | 1 | 1 | N/A | 1 | N/A | 1 | 0 | 13/14 | 93% |
| McCulloh. 2018 | 1 | 1 | 0 | | N/A | 1 | 0 | N/A | 0 | 1 | 1 | 1 | 1 | N/A | 1 | N/A | N/A | 1 | 9/12 | 75% |
| Mohammad. 2018 | 1 | 0 | 1 | | N/A | 1 | 0 | N/A | 0 | 1 | 1 | 1 | 1 | N/A | 1 | N/A | N/A | 1 | 9/12 | 75% |
| Padidar. 2019 | 1 | 1 | 1 | | N/A | 1 | 1 | N/A | 1 | 0 | 0 | 1 | 1 | N/A | 0 | N/A | N/A | 1 | 9/12 | 75% |
| Rath. 2018 | 0 | 1 | 1 | | N/A | 1 | 1 | N/A | 1 | 0 | 1 | 1 | 1 | N/A | 1 | N/A | N/A | 1 | 10/12 | 83% |
| Rath. 2019 | 1 | 1 | 1 | | N/A | 1 | 1 | N/A | 1 | 0 | 1 | 1 | 1 | N/A | 1 | N/A | N/A | 0 | 10/12 | 83% |
| Reid. 2013 | 1 | 1 | 1 | | 1 | 1 | 1 | 1 | 1 | 1 | 1 | 1 | 1 | 1 | 1 | 1 | 0 | 1 | 16/17 | 94% |
| Singh. 2017 | 0 | 0 | 1 | | N/A | 1 | 0 | N/A | 0 | 1 | 1 | 1 | 1 | N/A | 0 | N/A | N/A | 1 | 7/12 | 58% |
| Svedberg. 2019 |  |  |  | |  |  |  |  |  |  |  |  |  |  |  |  |  |  |  |  |
| Thabrew. 2019 | 1 | 1 | 1 | | 0 | 1 | 0 | N/A | 1 | 1 | 1 | 1 | 1 | 1 | 1 | 0 | N/A | 1 | 12/15 | 80% |
| Thabtah. 2018 | 0 | 0 | 1 | | N/A | 0 | 0 | N/A | 0 | 1 | 1 | 1 | 1 | N/A | 0 | N/A | N/A | 1 | 6/12 | 50% |
| Thompson. 2016 | 1 | 1 | 1 | | 0 | 1 | 0 | N/A | 1 | 1 | 1 | 1 | 1 | 1 | 1 | 0 | N/A | 1 | 12/15 | 80% |
| Valdes-Angues. 2018 | 1 | 1 | 1 | | N/A | 1 | 0 | N/A | 0 | 1 | 1 | 1 | 1 | N/A | 1 | N/A | N/A | 1 | 10/12 | 83% |
| van Karnebeek. 2012 |  |  |  | |  |  |  |  |  |  |  |  |  |  |  |  |  |  |  |  |
| Wang. 2017 |  |  |  | |  |  |  |  |  |  |  |  |  |  |  |  |  |  |  |  |

Items: (1) Is the hypothesis/aim/objective of the study clearly described? (2) Are the main outcomes to be measured clearly described in the Introduction or Methods section? (3) Are the characteristics of the patients included in the study described clearly? (4) Are the distributions of principal confounders in each group of subjects to be compared described clearly? (5) Are the main findings of the study described clearly? (6) Does the study provide estimates of the random variability in the data for the main outcomes? (7) Have the characteristics of patients lost to follow-up been described? (8) Have actual probability values been reported (for example, 0.035 rather than<0.05) for the main outcomes except where the probability value is less than 0.001? (9) Were the subjects asked to participate in the study representative of the entire population from which they were recruited? (10) If any of the results of the study were based on ‘data dredging’, was this made clear? (11) Were the statistical tests used to assess the main outcomes appropriate? (12) Were the main outcome measures used accurate (valid and reliable)? (13) Were the patients in different groups recruited from the same population? (14) Were study subjects recruited over the same period of time? (15) Was there adequate adjustment for confounding in the analyses from which the main findings were drawn? (16) Were losses of patients to follow-up taken into account? (17) Did the study have sufficient power to detect a clinically important effect where the probability value for a difference being due to chance is less than 5%?

Table S3. Quality assessment of studies included in the review using the National Institute for Health and Care Excellence (NICE) Quality Appraisal Checklist

|  | Theoretical approach | | Study design | Data collection | Trustworthiness | | | Analysis | | | | | | Ethics | Overall assessment |  |
| --- | --- | --- | --- | --- | --- | --- | --- | --- | --- | --- | --- | --- | --- | --- | --- | --- |
| Item | 1 | 2 | 3 | 4 | 5 | 6 | 7 | 8 | 9 | 10 | 11 | 12 | 13 | 14 | 15 |  |
| Alawna. 2019 |  |  |  |  |  |  |  |  |  |  |  |  |  |  |  |  |
| Binotti. 2019 |  |  |  |  |  |  |  |  |  |  |  |  |  |  |  |  |
| Boyce. 2019 | 1 | 1 | 1 | 1 | 1 | 1 | 1 | 1 | 0 | 0 | 1 | 1 | 1 | 1 | 2 |  |
| Den Boer. 2018 | 1 | 1 | 0 | 0 | 1 | 1 | 1 | 1 | 0 | 0 | 1 | 1 | 1 | 1 | 1 |  |
| Detsomboonrat. 2019 |  |  |  |  |  |  |  |  |  |  |  |  |  |  |  |  |
| Dexheimer. 2014 |  |  |  |  |  |  |  |  |  |  |  |  |  |  |  |  |
| Eikelboom. 2005 |  |  |  |  |  |  |  |  |  |  |  |  |  |  |  |  |
| Estai. 2016 |  |  |  |  |  |  |  |  |  |  |  |  |  |  |  |  |
| Finocchario-Kessler. 2015 | 1 | 1 | 0 | 1 | 1 | 1 | 1 | 0 | 1 | 0 | 1 | 1 | 1 | 0 | 1 |  |
| Franke. 2018 |  |  |  |  |  |  |  |  |  |  |  |  |  |  |  |  |
| Galvez. 2017 |  |  |  |  |  |  |  |  |  |  |  |  |  |  |  |  |
| Ginsberg. 2015 | 1 | 1 | 1 | 1 | 1 | 1 | 1 | 1 | 0 | 0 | 1 | 1 | 1 | 1 | 2 |  |
| Gregory. 2017 |  |  |  |  |  |  |  |  |  |  |  |  |  |  |  |  |
| Han. 2019 |  |  |  |  |  |  |  |  |  |  |  |  |  |  |  |  |
| Hashemi. 2017 |  |  |  |  |  |  |  |  |  |  |  |  |  |  |  |  |
| Heida, 2018 | 1 | 1 | 1 | 0 | 0 | 1 | 1 | 1 | 0 | 1 | 1 | 1 | 1 | 1 | 1 |  |
| Hussey. 2019 | 1 | 1 | 1 | 1 | 0 | 1 | 1 | 1 | 1 | 1 | 1 | 1 | 1 | 1 | 2 |  |
| Iorfino. 2017 |  |  |  |  |  |  |  |  |  |  |  |  |  |  |  |  |
| Jeong. 2019 | 1 | 1 | 1 | 1 | 0 | 1 | 1 | 1 | 1 | 1 | 1 | 1 | 1 | 1 | 2 |  |
| Jiam. 2017 | 1 | 1 | 1 | 0 | 0 | 0 | 1 | 1 | 0 | 0 | 1 | 1 | 1 | 0 | 1 |  |
| Kassam-Adams. 2019 |  |  |  |  |  |  |  |  |  |  |  |  |  |  |  |  |
| Kim. 2019 | 1 | 1 | 1 | 1 | 1 | 1 | 1 | 1 | 1 | 1 | 1 | 1 | 1 | 1 | 2 |  |
| Li. 2019 |  |  |  |  |  |  |  |  |  |  |  |  |  |  |  |  |
| March. 2018 | 1 | 1 | 1 | 1 | 0 | 1 | 1 | 1 | 1 | 1 | 1 | 1 | 1 | 1 | 2 |  |
| Matin. 2020 |  |  |  |  |  |  |  |  |  |  |  |  |  |  |  |  |
| McCulloh. 2018 | 1 | 1 | 0 | 1 | 0 | 1 | 0 | 0 | 0 | 1 | 1 | 1 | 1 | 0 | 1 |  |
| Mohammad. 2018 |  |  |  |  |  |  |  |  |  |  |  |  |  |  |  |  |
| Padidar. 2019 |  |  |  |  |  |  |  |  |  |  |  |  |  |  |  |  |
| Rath. 2018 | 1 | 0 | 1 | 0 | 1 | 1 | 1 | 1 | 1 | 1 | 1 | 0 | 1 | 0 | 1 |  |
| Rath. 2019 |  |  |  |  |  |  |  |  |  |  |  |  |  |  |  |  |
| Reid. 2013 |  |  |  |  |  |  |  |  |  |  |  |  |  |  |  |  |
| Singh. 2017 |  |  |  |  |  |  |  |  |  |  |  |  |  |  |  |  |
| Svedberg. 2019 | 1 | 1 | 0 | 0 | 0 | 1 | 1 | 1 | 1 | 1 | 1 | 1 | 1 | 1 | 1 |  |
| Thabrew. 2019 | 1 | 1 | 1 | 1 | 0 | 0 | 1 | 1 | 1 | 1 | 1 | 1 | 1 | 1 | 2 |  |
| Thabtah. 2018 |  |  |  |  |  |  |  |  |  |  |  |  |  |  |  |  |
| Thompson. 2016 |  |  |  |  |  |  |  |  |  |  |  |  |  |  |  |  |
| Valdes-Angues. 2018 | 1 | 1 | 0 | 1 | 0 | 1 | 1 | 1 | 0 | 1 | 1 | 1 | 0 | 0 | 1 |  |
| van Karnebeek. 2012 | 1 | 0 | 1 | 1 | 0 | 1 | 0 | 0 | 0 | 0 | 1 | 1 | 1 | 0 | 1 |  |
| Wang. 2017 | 1 | 1 | 1 | 1 | 1 | 1 | 0 | 1 | 1 | 1 | 1 | 1 | 1 | 0 | 2 |  |
